# *Angiostrongylus cantonensis* in North African hedgehogs as vertebrate hosts, Mallorca, Spain, October 2018

**DOI:** 10.2807/1560-7917.ES.2019.24.33.1900489

**Published:** 2019-08-15

**Authors:** Claudia Paredes-Esquivel, Jessica Sola, Sofía Delgado-Serra, Miguel Puig Riera, Nieves Negre, Miguel Ángel Miranda, José A Jurado-Rivera

**Affiliations:** 1 Applied Zoology and Animal Conservation Group, University of the Balearic Islands, Palma de Mallorca, Spain; 2 Centre de Recuperació de Fauna Silvestre de les Illes Balears (COFIB), Mallorca, Spain; 3 Laboratory of Genetics, University of the Balearic Islands, Palma de Mallorca, Spain

**Keywords:** Angiostrongylus cantonensis, emerging zoonosis, Spain, hedgehogs, eosinophilic meningoencephalitis, lungworms, biosentinel

## Abstract

In October 2018, two *Atelerix algirus* hedgehogs were admitted to the Wildlife Rehabilitation Hospital in Mallorca (Balearic Islands, Spain) with signs of acute neurological disease. Necropsy detected immature, fully developed nematodes in the subarachnoid space of both hedgehogs, including a gravid female worm. DNA-based molecular tools confirmed the nematode as *Angiostrongylus cantonensis*, an important aetiological agent of eosinophilic meningitis in humans. So far this zoonotic parasite in has not been reported in western European wildlife.

We report the presence of the rat lungworm, *Angiostrongylus cantonensis*, in two *Atelerix algirus* (North African) hedgehogs taken from two different localities on Majorca (Balearic Islands, Spain). This report highlights the importance of using wildlife hosts as biosentinels of potentially emerging zoonosis

## Clinical and pathological findings in the hedgehogs

**Figure 1:**
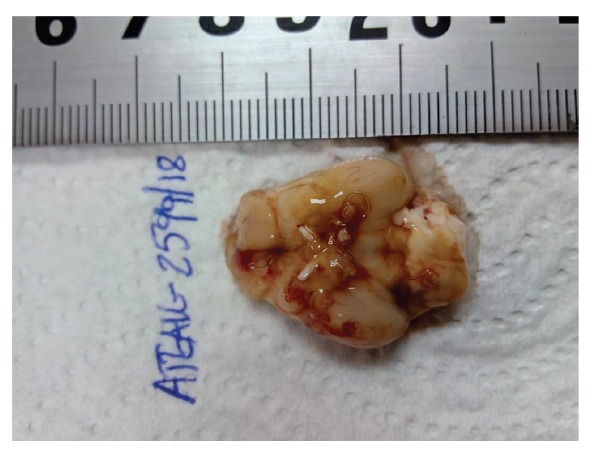
Ventral view of the brain of a male *Atelerix algirus* hedgehog, Mallorca, Spain, 2018

In October 2018, two adult (one male and one female) *A. algirus* hedgehogs were admitted to the Wildlife Rehabilitation Centre (COFIB) in Mallorca (Balearic Islands, Spain) from two different locations on the island. The animals showed signs of an acute central nervous system disorder. Symptoms included pelvic limb ataxia, atonia, posterior paresis of thoracic limbs and behavioural decay. Clinical manifestations progressed over a few days and hedgehogs were euthanised (female 1 day and male 10 days after admittance) to avoid any further suffering. Necropsy revealed the presence of nematode worms in the subarachnoid space of the brain in both hedgehogs. The female hedgehog was infected with a single nematode larva (11.1 mm long) and presented no perceptible lesions of the pia mater. The male hedgehog was infected with five fully developed nematodes, including a gravid female and one immature nematode; the pia mater exhibited lesions compatible with multifocal haemorrhage (Figure 1).

Several nematode specimens and lesions compatible with multifocal haemorrhage can be observed.

**Figure 2:**
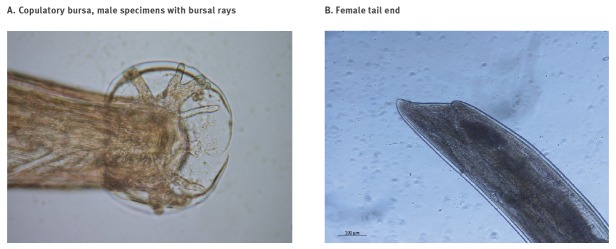
*Angiostrongylus cantonensis* views of (A) copulatory bursa of male specimens supported by bursal rays and (B) a female tail end, Spain, 2018

Adult specimens extracted from the male hedgehog measured 15–17 mm in length. Morphological examination following the dichotomous keys by Kinsella [1] suggested the nematodes were *A. cantonensis*. Male nematodes showed a copulatory bursa (Figure 2A) and gravid females showed a ‘barber’s pole’ appearance, blood-containing intestine and the typical morphology of the tail (Figure 2B).

Spiculae are not evident since voucher specimens have not been treated with clearing solutions.

## Molecular identification of nematode species

**Figure 3:**
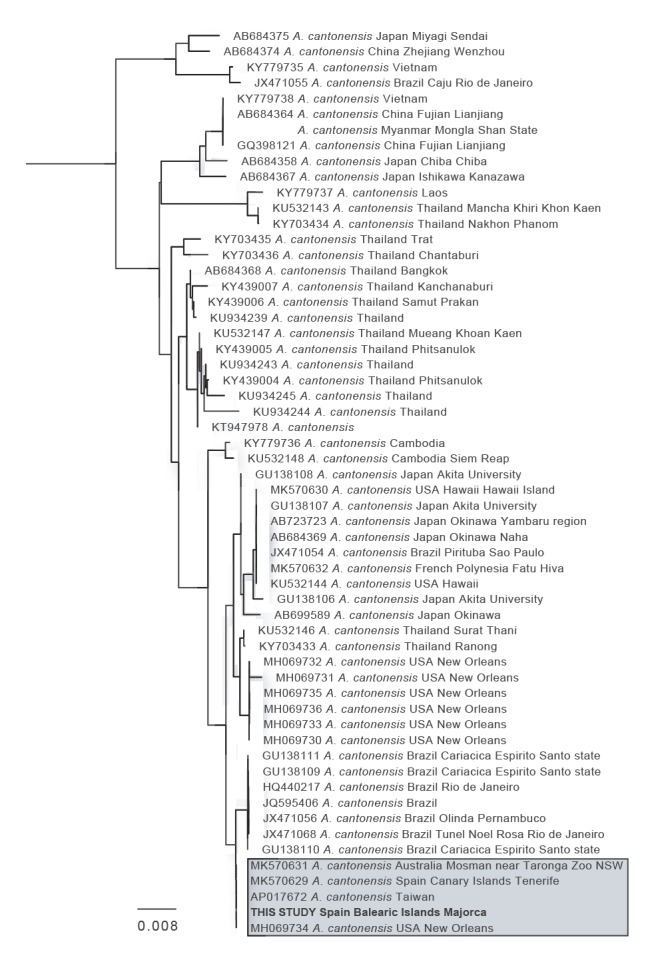
Neighbour-joining tree based on the sequence from the *Cytochrome Oxidase I* gene region from three nematode specimens, Spain, 2018

The species identity of the nematodes was confirmed by PCR and sequencing of the *Cytochrome Oxidase*/gene region from three specimens, using primers COI F: 5’- TTTTTTGGGCATCCTGAGGTTTAT -3’ and COI R: 5’- TAAAGAAAGAACATAATGAAAATG 3’ [2]. PCR reactions were prepared in a total volume of 25μL containing 17.2μL of water, 2.5μL of 10 x Bioline PCR buffer, 1.75μL of MgCl2 (50mM), 1μL of dNTP mix (10mM total), 0.5μL of each primer (100μM), 0.5μL of BSA (20mg/mL; New England BioLabs, Hitchin, Hertfordshire, United Kingdom), 0.05μL of Bioline Taq DNA polymerase and 1μL of template DNA. Reactions were incubated at 94 °C for 5 mins, followed by 35 cycles at 94 °C for 30s, 48 °C for 30s and 72 °C for 45s and a final extension step at 72 °C for 10 mins. PCR products were visualised on 1.5% agarose gel electrophoresis stained with ethidium bromide and purified using MSB Spin PCRapace (Invitek, Berlin, Germany) for subsequent sequencing using the BigDye Terminator Cycle Sequencing kit (Applied Biosystems, Foster City, California, United States). Three PCR amplifications were conducted on individual specimens. All three sequenced samples resulted in the same *COI* haplotype. This DNA sequence was blasted against the GenBank database and the top 57 hits corresponded with *COI* sequences of *A. cantonensis*, with the first four alignments yielding a 100% identity match with specimens from Australia, Tenerife, Taiwan and New Orleans (US), respectively (Figure 3). The sequence has been submitted to the GenBank website (accession number MN227185) and two adult nematode specimens and the DNA extractions used in this study have been kept as vouchers at -80 °C in the laboratory of zoology of the University of the Balearic Islands for further investigation.

The most similar sequences retrieved from GenBank after using the haplotype from the Balearic Islands in a BLAST search were used to generate a Neighbour-joining tree by selecting the ‘distance tree of results’ option implemented in the BLAST server. The geographical source of the sequences has been appended to the taxa names when available.

## Discussion

Angiostrongyliasis is an emerging zoonosis [3] caused by the metastrongyloid nematode *Angiostrongylus cantonensis*. Rats are the definitive hosts of this parasitic nematode, while birds and mammals act as accidental hosts. Infection typically occurs after vertebrates ingest intermediate hosts (snails and slugs) or paratenic hosts (freshwater shrimps, crabs, flatworms and frogs). Gastropod mucus may also be involved in the transmission cycle [4]. Severe clinical manifestations have been reported in vertebrate hosts, due to the parasite’s tropism to the central nervous system [5]. In humans, it is a leading cause of parasitic eosinophilic meningitis [6]. Angiostrongyliasis eosinophilic meningitis (AEM) is difficult to diagnose [3] and requires a high degree of clinical suspicion since eosinophilia may not be initially observed in peripheral blood [7] and serology may be misleading [8]. In areas where angiostrongyliasis is not endemic, AEM may thus be overlooked [9].

Human angiostrongyliasis is endemic to the Caribbean, Pacific Islands and Asia and has been shown to spread to temperate and sub-tropical regions [9], with about 3,000 cases reported in 30 countries [3]. Infected rats carried by ships have been suggested as the main agents of parasite expansion in the Asia-Pacific region after World War II [10] and may still be implicated in its further spread. Since the detection of angiostrongyliasis in rats in the Atlantic island of Tenerife (Canary Island, Spain) further spread to Europe has been anticipated by some [11]. However, to date, only a single autochthonous human case of angiostrongyliasis has been reported from France in 2016. Imported food could not be ruled out as a possible source of contamination, local active transmission could not be confirmed [12].

Hedgehogs are zoonotic agents of various parasitic diseases [13], but they have never been shown to act as reservoir hosts for parasites of the genus *Angiostrongylus* [5]. We had previously observed hedgehogs with clinical manifestations compatible with acute neurological disease, but necropsy did not warrant examination of parasitic nematodes in the brain, as no previous reports of nematodes had been previously reported in hedgehogs. The North African hedgehog, is widely distributed in the western Mediterranean Basin and in the Canary Islands. It is the most common mammal species hospitalised at the COFIB and its diet mainly consists of insects and snails. Most hedgehog fatalities at the centre are due to another gastropod-borne lungworm, *Crenosoma striatum* [14].

The presence of an adult gravid female nematode in the hedgehog’s brain was an interesting finding, as in accidental hosts only the sub-adult (fifth) stage has been found in the central nervous system [15]; moreover, our specimens were slightly smaller than those found in the pulmonary arteries of rats (size range 18.5–33) [16]. We did not search for adult worms in the pulmonary arteries of hedgehogs and we did not conduct any survey in rats in the island. These are the main limitations of this study. Further investigations should clarify the role of hedgehogs in the life cycle of *A. cantonensis*.

The Balearic hedgehog populations are geographically isolated and two specimens from different locations were infected suggesting that active transmission of *A. cantonensis* is occurring on the island; this is the first report of *A. cantonensis* infecting western European wildlife. Majorca has strong connection by sea and air with mainland Europe and millions of tourists visit the island each year (e.g. 16.5 million in 2018) [17], this and the ubiquity of rats and snails on the island could facilitate a spread of *A. cantonensis*.

Snails are an important part of the Majorcan cuisine and they are commercialised in heliciculture farms on the island or inhabitants can collect wild snails from open fields. It is therefore, beneficial to determine which intermediate host species are involved in the transmission of *A. cantonensis.* A previous r*e*port in Tenerife indicated that the garden snail (*Cornu aspersum*) and Mediterranean snail (*Theba pisana*) are competent vectors of *A. cantonensis* [18]; these species are also present in Balearic Islands. The best way to prevent human infections is by not eating raw or undercooked snails or freshwater shrimps or other paratenic hosts. However, pieces of snails accidently eaten in inadequately washed/cooked vegetables have been associated with possible cases of eosinophilic meningitis [4]. The risk of human infection could be avoided if control measures e.g. wearing gloves when handling snails/slugs, washing hands are undertaken [19]. Additional measures that could be taken are increasing public awareness of *A. cantonensis* and surveillance of vectors and vertebrate hosts in endemic areas.

## Conclusions

This report highlights the importance of using wildlife hosts as biosentinels for possible emerging zoonotic infections and the need to consider *A. cantonensis* as potential aetiological agent of eosinophilic meningoencephalitis within western Europe.
